# Reversible and Rapid Transfer-RNA Deactivation as a Mechanism of Translational Repression in Stress

**DOI:** 10.1371/journal.pgen.1003767

**Published:** 2013-08-29

**Authors:** Andreas Czech, Sandra Wende, Mario Mörl, Tao Pan, Zoya Ignatova

**Affiliations:** 1 Institute of Biochemistry and Biology, University of Potsdam, Potsdam, Germany; 2 Institute for Biochemistry, University of Leipzig, Leipzig, Germany; 3 Department of Biochemistry and Molecular Biology, University of Chicago, Chicago, United States of America; Cornell University, United States of America

## Abstract

Stress-induced changes of gene expression are crucial for survival of eukaryotic cells. Regulation at the level of translation provides the necessary plasticity for immediate changes of cellular activities and protein levels. In this study, we demonstrate that exposure to oxidative stress results in a quick repression of translation by deactivation of the aminoacyl-ends of all transfer-RNA (tRNA). An oxidative-stress activated nuclease, angiogenin, cleaves first within the conserved single-stranded 3′-CCA termini of all tRNAs, thereby blocking their use in translation. This CCA deactivation is reversible and quickly repairable by the CCA-adding enzyme [ATP(CTP):tRNA nucleotidyltransferase]. Through this mechanism the eukaryotic cell dynamically represses and reactivates translation at low metabolic costs.

## Author Summary

Adequate reprogramming of metabolic activities by environmental stress or suboptimal growth conditions is crucial for cell survival. Cells employ a remarkable diversity of processes to maintain its homeostasis at all levels of gene expression, including chromatin remodeling, mRNA expression and degradation, translation and protein degradation. Each of these processes shapes cell response at different time scales. In this study, we analyzed the cellular response to oxidative stress at the level of translation. Translation, as one of the most downstream processes in gene expression, provides the necessary plasticity for immediate changes of cellular activities. Using high-sensitive approaches to probe the structural integrity of cellular tRNAs, we show that upon exposure to oxidative stress tRNAs are rapidly deactivated by a cleavage within their ubiquitous, single-stranded 3′-CCA termini by oxidative stress-activated nuclease, angiogenin. The CCA-ends deactivation is reversible and quickly repairable by a ubiquitous enzyme, CCA-adding enzyme, whose natural function is to attach post-transcriptionally the CCA overhang to the 3′-termini of all tRNAs in an mRNA template-independent manner. We propose that this is a mechanism to dynamically repress and reactivate translation at low metabolic costs.

## Introduction

Environmental stress or suboptimal growth conditions reduce cell viability and put cells at risk. Cells maintain their internal homeostasis by adequate reprogramming of metabolic activities at all levels of gene expression, including chromatin remodeling, mRNA expression and degradation, translation and protein degradation. Given the considerable time needed to activate new genes and/or *de novo* synthesize mRNA, the translation of existing mRNAs provides the necessary plasticity for the cell to selectively and rapidly respond to stress [1], [2]. Translation is divided into three distinct phases: initiation, elongation and termination. Translation initiation, as a rate-limiting process, is a major point to reprogram translation in response to stress [3], [4]. A key mechanism to repress translation initiation is the phoshorylation of the alpha-subunit of translation initiation factor 2 (eIF2) by stress-activated kinases [5], [6]. However, a sizeable set of cellular mRNAs are initiated in an eIF2-independent manner, which allows for escaping the global kinase-dependent inhibition of translation initiation [3], [4]. It remains elusive, which alternative mechanisms the cell employs to regulate translation during adverse environmental stress.

Transfer RNAs (tRNAs) enter ribosome-mediated protein biosynthesis in a translationally competent state, which includes post-transcriptional modifications at various positions, including the anticodon loop, and the presence of an intact single-stranded CCA-sequence at the 3′-terminus that is required for amino acid attachment by the corresponding aminoacyl-tRNA-synthetase [7]. The CCA ends are generated and maintained by the CCA-adding enzyme [8]. Some bacteria carry tRNA genes encoding CCA termini, thus the CCA-adding enzyme is primarily involved in repairing damaged CCA ends in these organisms [9]. In contrast, all eukaryotic tRNA genes lack the CCA ends and the role of the CCA-adding enzyme is to attach post-transcriptionally the CCA overhang to the 3′-termini of all tRNAs [8], [10], [11]. The functional repertoire of the CCA-adding enzyme has been expanded by its recently discovered role in the quality control of hypo-modified tRNAs [12].

Mature, translationally competent tRNAs are very stable under normal growth conditions, with a half-life of approximately one to several hours [13]. However, environmental changes dynamically modulate the concentration of the tRNA pool. Some tRNAs are cleaved in the anticodon loop in response to various environmental stress factors (e.g., oxidative stress, heat shock or ultraviolet irradiation) [14], [15], [16], [17]. The endonucleolytic tRNA cleavage is a conserved feature in higher eukaryotes. Thereby, two tRNA-halves (designated 5′- and 3′-tiRNAs) are generated by a ubiquitously expressed enzyme, angiogenin [18]. This cleavage, however, does not significantly reduce the level of mature tRNAs, which implies that tiRNAs may rather act as a signal transducer to modulate translation of specific mRNAs, than to globally repress translation [18]. Furthermore, in response to external stimuli, retrograde translocation of mature tRNAs to the nucleus[19] or selective charging of different tRNA isoacceptors [20] transiently alter the pool of translationally active tRNAs in the cytoplasm. Consequently, these stress-induced alterations in the tRNA concentration will decrease the amount of ternary complex (that is, the complex of charged tRNA with the GTP-loaded elongation factor). However, the primary mechanism, that triggers a general inhibition of translation elongation during stress, remains surprisingly elusive.

Here, using high-sensitive approaches to probe the structural integrity of cellular tRNAs, we show that upon exposure to oxidative stress all tRNAs are rapidly deactivated by a cleavage within their 3′-CCA termini by oxidative stress-activated nuclease, angiogenin. Since 3′-CCA ends are ubiquitous for all tRNAs, angiogenin-induced deactivation of tRNAs provides a means for global repression of translation at the level of elongation. On a much slower scale, at longer times of exposure to stress, some tRNAs are also cut in their anticodon. The CCA ends deactivation is reversible and quickly repairable by the CCA-adding enzyme. We propose that this is a mechanism to dynamically repress and reset translation at low metabolic costs.

## Results

### 3′-CCA ends of tRNAs are first cleaved by oxidative stress

**Figure 1:**
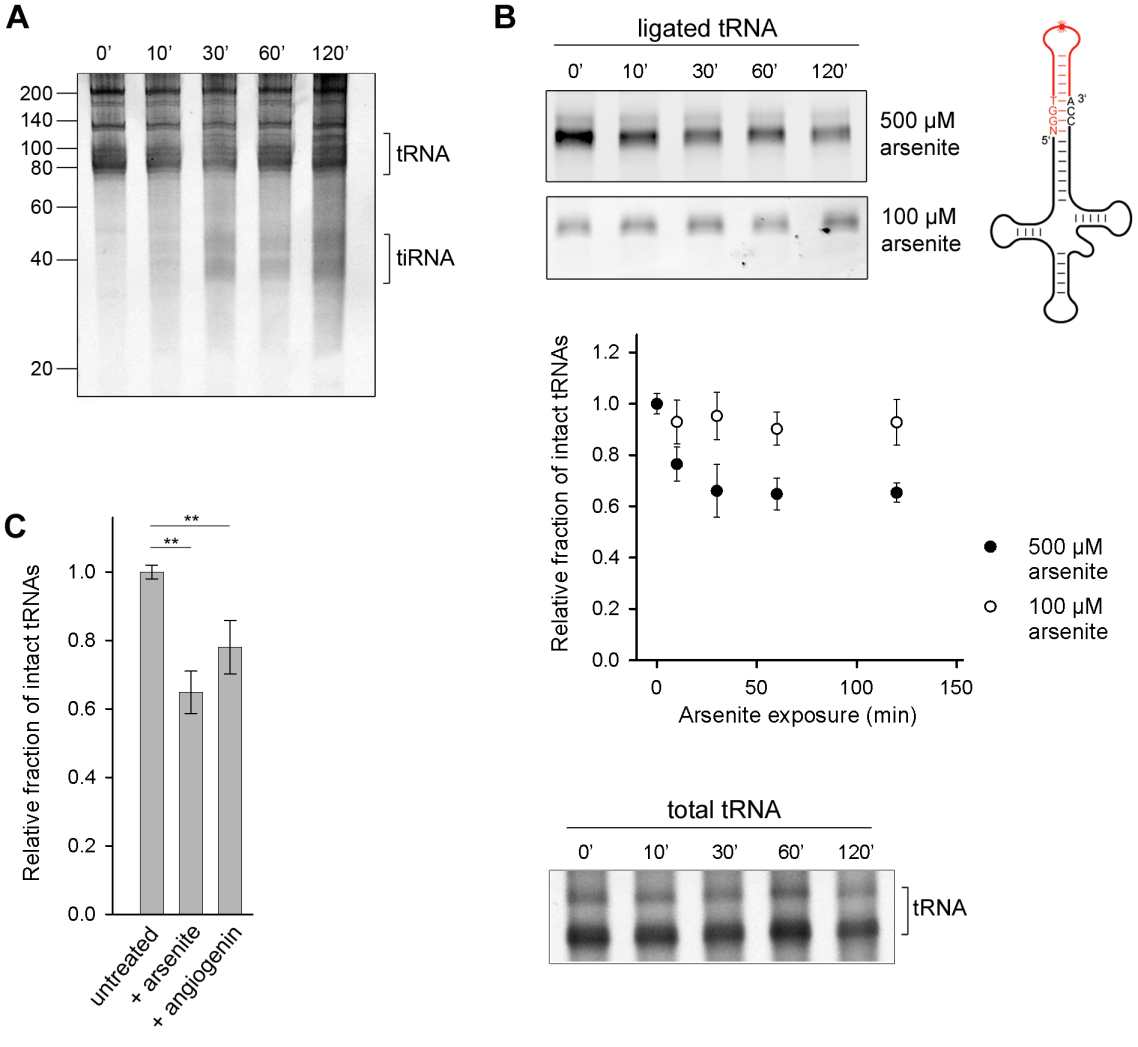
Oxidative stress-mediated tRNA cleavage in HeLa cells. (A) Minor fraction tiRNAs are generated by longer exposure to 500 µM arsenite (>30 min). The numbers on the left denote the DNA ladder in nt. (B) Arsenite alters the integrity of the 3′-CCA end of full-length tRNAs in a dose-dependent manner. The amount of tRNAs with intact CCA ends was analyzed by their ability to ligate to a fluorescently labeled oligonucleotide (schematic inset) which forms a loop and binds only intact 3′-CCA ends (upper two gels). The intensity of tRNAs with intact CCA termini was quantified from the gels, normalized to the total tRNA amount at each time point and presented as relative values ± SEM (from three independent experiments) to the amount of initial, untreated sample which was set as 1.0. The amount of the total tRNA remained almost unchanged when cells were exposed to arsenite (500 µM) and visualized by SYBR Green (bottom gel marked as total tRNA). (C) Increase of the cellular concentration of angiogenin alters the 3′-CCA integrity of tRNAs. Angiogenin was upregulated by ectopic expression under the control of a CMV promoter for 8 h (+angiogenin). The sample representing arsenite stress (+arsenite) corresponds to the 60-min data point at 500 µM arsenite in panel (B) and is used for comparison. The intensity of tRNAs with intact CCA termini was the quantified as described for panel (B). ** for *p*<0.01.

Angiogenin, the nuclease that endonucleolytically cleaves tRNAs during oxidative stress, is constitutively expressed, but kept inactive through an inhibitor RNH1 [18]. Oxidative stress dissociates the inhibitor and activates angiogenin [21]. To investigate the susceptibility of the cellular tRNAs to angiogenin-mediated cleavage, we exposed confluent HeLa cells to arsenite which elicits oxidative stress and activates angiogenin. A small amount of tiRNAs was generated, but only at prolonged exposure to arsenite (>30 min) (Figure 1A). Next, we used tRNA microarrays [22] with immobilized oligonucleotide probes complementary to the full-length tRNA sequences to determine the susceptibility of each tRNA species to angiogenin. Only a subset of all tRNAs bearing a CA sequence in the anticodon loop was predominantly cleaved into tiRNAs; a minor fraction with UA or GC motifs in the anticodon loop was also cleaved (Figure S1A). This cleavage pattern mirrors the substrate specificity of angiogenin: it targets single-stranded ribonucleic acid sequences with 10–30-fold higher preference for CA over UA [23] and 3-fold higher for CA over CG [24]. While there is a large variability in the composition of the anticodon loops of all tRNAs and only a fraction of tRNAs possesses a CA-motif in the anticodon loop (Figure S1B), we realized that the ubiquitous, single-stranded 3′-CCA sequence post-transcriptionally attached to all eukaryotic tRNAs [8], bears the strongest angiogenin recognition motif, the CA motif. To investigate whether the 3′-CCA ends can be targeted by angiogenin upon exposure to oxidative agents, we used enzymatic ligation of a fluorescent stem-loop oligonucleotide that complementary pairs only to the intact 3′-CCA end of tRNA (Figure 1B, schematic inset). The fluorescent signal, which is proportional to the amount of tRNAs with intact 3′-CCA ends, decreased noticeably in conditions of severe oxidative stress while the total tRNA amount remained relatively constant (500 µM arsenite; Figure 1B), consistent with the idea that the 3′-CCA ends of tRNAs are primary substrates of angiogenin. Oxidative stress altered the structural integrity of the 3′-CCA termini of tRNAs in a dose-sensitive manner; at lower dose of stress (100 µM arsenite) much smaller fraction of tRNAs than at high stress dose (500 µM arsenite) was unable to ligate the fluorescent oligonucelotide (Figure 1B). To our surprise, the removal of the 3′-CCA ends of the tRNAs occurred on a much faster time scale (Figure 1B) compared to the appearance of the tiRNA fragments (Figure 1A).

To confirm that angiogenin cleaves the 3′-CCA termini of tRNAs upon exposure to arsenite, we upregulated the level of aniogenin and analyzed the integrity of the 3′-CCA ends using the fluorescent oligonucleotide-ligation approach. A small increase in the cellular level of angiogenin led to a noticeable enrichment of tRNAs with cleaved 3′-CCA termini, confirming its role in the oxidative stress-mediated cleavage of the 3′-CCA ends (Figure 1C). Note that angiogenin can be only moderately upregulated for short expression times (Figure S2A); longer expression perturbs the vitality of the cell. The ectopically enhanced levels of angiogenin, a fraction of it might be additionally deactivated by the excess of the RNH1 inhibitor in the cell, is most likely far below the concentration of stress-activated angiogenin, thus the effect of arsenite-induced 3′-CCA end cleavage (+arsenite) was much stronger (Figure 1C).

**Figure 2:**
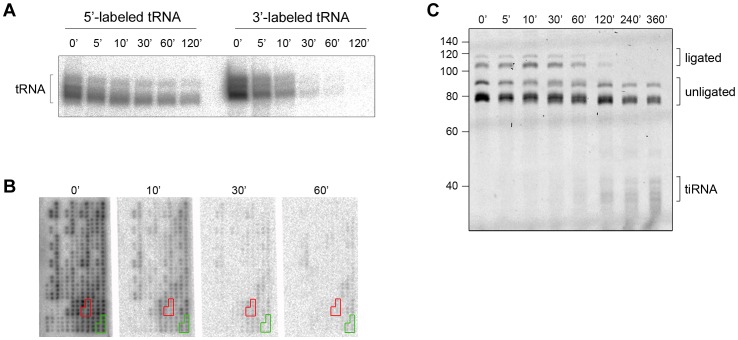
The CCA sequence at the 3′-termini of all tRNAs is first cleaved by angiogenin *in vitro*. (A) Angiogenin (1 µM) digestion of total HeLa tRNA radioactively labeled at either 5′- or 3′-end. (B) 3′-radioactively labeled HeLa tRNAs treated with 1 µM angiogenin for different times and their intact 3′-termini were visualized with tRNA macroarrays. Only tRNAs (or fragments of them) with intact 3′-ends are visible on the microarrays. Two exemplary tRNAs (Ala-IGC, green and Gln-yTG, red) are marked. Probes for each tRNA are arranged in clusters of six replicates. (C) Analysis of the integrity of the 3′-CCA end of full-length tRNAs with the specific oligonucleotide-ligation approach after angiogenin (0.2 µM) treatment for various times. The gel was visualized with SYBR Green. Note, to better resolve the kinetics of cleavage we decreased the concentration of angiogenin to 0.2 µM; thus the time points here are not directly comparable with the time points in panels A, B. The numbers on the left denote the DNA ladder in nt. Multiple bands for tiRNAs, full-length tRNAs, ligated and unligated tRNAs are detected (panels A and C) due to the natural variations in tRNAs length.

Intrigued by the different time scales of the stress-induced alterations of cellular tRNAs, we next analyzed the kinetics of 3′-CCA end cleavage and tiRNA generation *in vitro*. Total tRNA was isolated from confluent, non-stressed HeLa cells, radioactively labeled at their 5′- or the 3′-end and subsequently subjected to angiogenin treatment. Strikingly, while 5′-labeled tRNAs are still visible at 120 min of incubation with angiogenin, the 3′-labeled tRNAs completely disappeared after 30 min (Figure 2A). The fast decay of the signal of the 3′-labeled full length tRNAs than the 5′-labeled tRNAs (Figure 2A) is consistent with a preferred and much faster cleavage in the 3′-CCA termini of the tRNAs. All tRNAs were equally sensitive to angiogenin cleavage at the 3′-CCA termini; the signal for all 3′-radioactively labeled tRNAs decayed almost simultaneously during the angiogenin treatment (Figure 2B and S3). In the fluorescent oligonucleotide-ligation approach (Figure 1B, schematic inset), we observed a clear progressive decrease of the yield of the oligonucleotide ligated to the 3′-CCA end of the tRNAs upon angiogenin treatment (Figure 2C). Notably, fragments migrating at the height of the tiRNAs appeared much later (Figure 2C), suggesting that angiogenin degraded the 3′-CCA ends more rapidly than it cleaved tRNAs in the anticodon loop, thus recapitulating the observations in HeLa cells (Figure 1). To define the exact cleavage site in the 3′-CCA end, we used internally radioactively labeled variants of tRNA^Phe^(GAA) with intact 3′-CCA and truncated 3′-CC end. tRNA^Phe^(GAA) lacks the CA motif in the anticodon loop and hence, only the 3′-CCA terminus is susceptible to angiogenin cleavage. Angiogenin cleaved endonucleolytically within the 3′-CCA motif between the C and A nucleotide and removed exclusively the adenosine residue (Figure S4), implying a high CA-dependent endonucleolytic activity of angiogenin.

### Cleaved 3′-CCA ends are repaired by the CCA-adding enzyme

While *in vitro* all tRNAs lost their 3′-ends after 10 min, as evidenced by almost complete signal extinction of the 3′-labeled full-length tRNAs (Figure 2A), *in vivo* the signal plateaued at about 60% of the initial signal intensity (Figure 1B). Translationally competent tRNAs are aminoacylated and complexed with elongation factor EF1α, which may protect tRNAs from angiogenin cleavage. Aminoacylation *per se* did not influence angiogenin cleavage (Figure S5). The crystal structure of the ternary complex from *Thermus aquaticus* indicates that EF1α contacts only the phosphate groups of tRNA bases 73–75 [25]. [Note, C75A76 is endonucleolytically targeted by angiogenin]. Thus, the elongation factor EF1α may marginally interfere with the angiogenin binding and partly protect the aminoacyl-tRNA.

**Figure 3:**
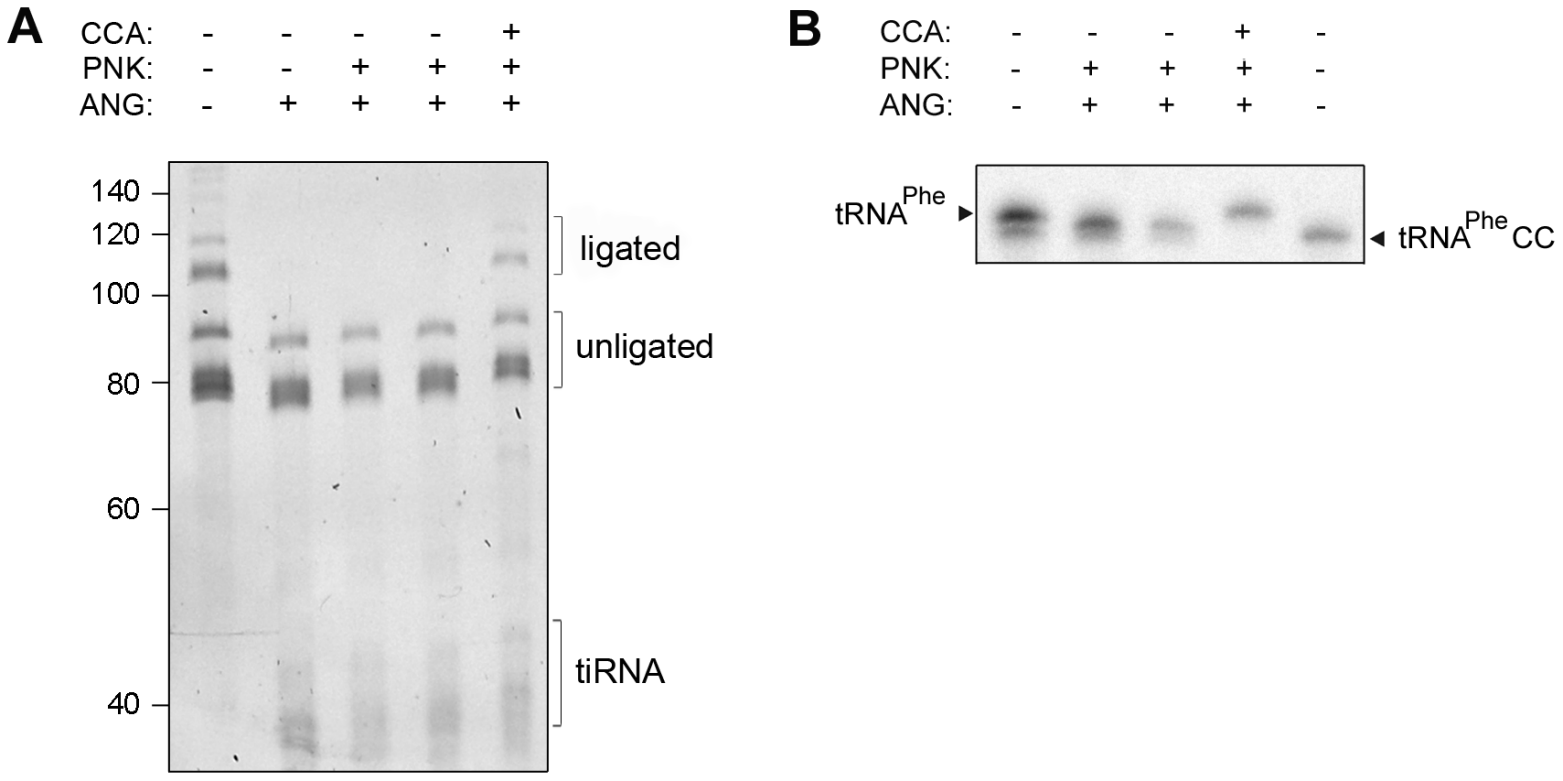
Human CCA-adding enzyme is able to repair damaged CCA ends of tRNAs. (A) Total HeLa tRNAs and (B) internally radioactively labeled yeast tRNA^Phe^ were incubated successively with angiogenin and human CCA-adding enzyme. Subsequently to the angiogenin treatment (4 h), T4 polynucleotide kinase (PNK) (45 min) was added which converts the 2′,3′-cyclophosphate ends [42] generated by the angiogenin cleavage to free 3′OH. After purification tRNAs were subjected to treatment with the CCA-adding enzyme (30 min). The 3′-CCA end integrity of the HeLa tRNAs was determined with the fluorescent oligonucleotide (Figure 1A, schematic inset). tRNA^Phe^ lacking the terminal 3′-adenosine (tRNA^Phe^CC) served as a control. The numbers on the left denote the DNA ladder in nt.

In cells, the CCA-adding enzyme repairs the partially degraded 3′-CCA ends of tRNAs without a nucleic acid template and highly discriminates between adding cytidine at position 75 and adenosine at position 76 [8], [10], [11]. Thus, we hypothesized that the lower amount of tRNAs with deactivated CCA termini in HeLa cells (Figure 1B), compared to the *in vitro* angiogenin treatment (Figure 2A), might represent a steady-state equilibrium between the angiogenin-mediated cleavage and simultaneous repair by the CCA-adding enzyme whose activity remained unchanged upon the arsenite treatment (Figure S4C). To determine the effect of these two opposing processes, total HeLa tRNAs were successively treated with angiogenin and human CCA-adding enzyme. Indeed, the CCA-adding enzyme repaired the CCA termini (Figure 3A) by adding the cleaved adenosine (Figure 3B), implying that stress-damaged 3′-CCA ends of tRNAs can be easily repaired and tRNAs are converted back to translationally-competent species. The angiogenin-catalyzed endonucleolytic cleavage of the CA motif results in a 3′-terminal 2′,3′-cyclic phosphate at the cytosine residue. Thus, prior to treatment with CCA-adding enzyme HeLa tRNAs (Figure 3A) or single tRNA^Phe^CC were treated with T4 polynukleotide kinase (PNK). In the cell, the 3′-end cyclization products are quickly hydrolyzed to 3′OH by 2′,3′-cyclic 3′-phosphodiesterases [26], [27].

An attempt to reduce the cellular concentration of the CCA-adding enzyme was unsuccessful: even though *de novo* synthesis of the enzyme was significantly inhibited by targeting its mRNA with specific siRNA probe (Figure S2B), the concentration of the mature CCA-adding enzyme remained unchanged (Figure S2C). As the CCA-adding activity is essential for cell viability, an intrinsic robustness of this enzyme has the advantage of maintaining a constant function and permitting a prompt stress response.

### 3′-CCA ends deactivation represses translation elongation

**Figure 4:**
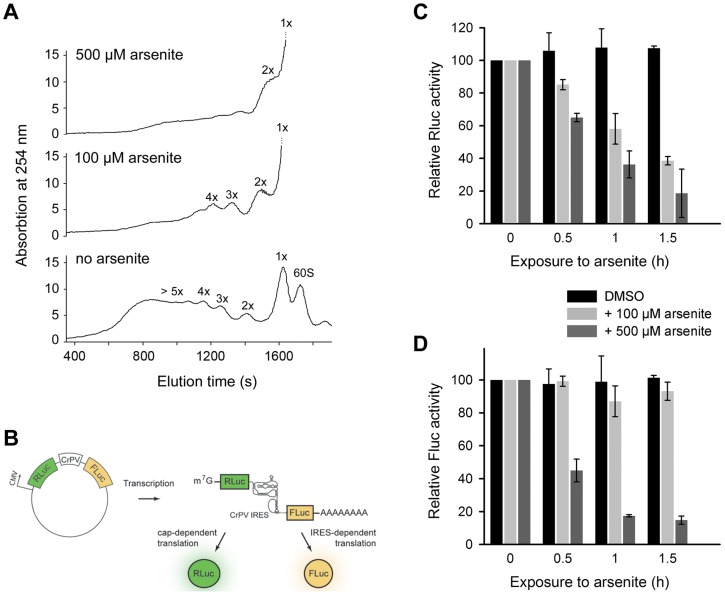
Scanning-independent translation initiation is less influenced by low dose oxidative stress. (A) Polysomal profiles of untreated or arsenite treated HeLa cells. (B) Schematic of the plasmid used to monitor cap-dependent and IRES-dependent translation initiation. Inhibition of cap-dependent, Rluc (C) or IRES-mediated, Fluc (D) translation upon exposure to oxidative stress. HeLa cells expressing the bicistronic construct encoding Rluc under the CMV-promoter (scanning-dependent translation) and Fluc under the CrPV-IRES (non-scanning controlled translation) were exposed to different arsenite concentrations for various times. Addition of DMSO to the cells served as a control. Data in (C) and (D) ± SEM are normalized to the first data point for which the activity was set as 100.

The 3′-CCA ends are indispensable for tRNA aminoacylation and subsequently for translation. What is the effect of angiogenin-induced deactivation of the 3′-CCA termini of cellular tRNAs on protein translation? Exposure of HeLa cells to acute oxidative stress (500 µM arsenite) altered the polysomal profile and shut down translation (Figure 4A). Importantly, at low arsenite concentration (100 µM) the cells retained some translation activity, detectable as a considerable polysomal fraction (Figure 4A). The most potent inhibition of translation is mediated by eIF2α phosphorylation upon oxidative stress via haem-regulated inhibitor kinase (HRI), which represses translation of mRNAs with scanning- or cap-dependent translation initiation [3]. By contrast, a sizeable subset of genes are translated through a cap-independent mechanism: internal ribosome-entry sites (IRES) direct translation initiation without the aid of canonical initiation factors and initiator Met-tRNA [28]. We hypothesized that cap-dependent translation will be influenced at much lower arsenite concentrations compared to mRNAs with scanning-independent initiation; the combined effect of oxidative stress on eIF2α phosphorylation and the deactivation of the 3′-CCA ends of all tRNAs will have much higher impact on mRNAs initiated in a scanning-dependent manner. In contrast, in the IRES-initiated translation, as only the 3′-CCA-end inactivation should play a role the effect should be less pronounced. We therefore tested the effect of two arsenite concentrations, representing severe (500 µM) and moderate (100 µM) oxidative stress using bicistronic mRNA encoding renilla luciferase (Rluc), initiated in a cap-controlled manner, and firefly luciferase (Fluc), initiated via cricket paralysis virus IRES (CrPV-IRES) (Figure 4B), an IRES sequence described to confer translation independent of any initiation factor [29]. At a low arsenite concentration (100 µM), the Fluc activity remained at >80%, while Rluc activity progressively decreased, indicating much potent inhibition of cap-dependent initiation compared to IRES-dependent initiation (Figure 4C,D). At a high arsenite concentration (500 µM), however, a similar decrease for both Rluc and Fluc activity was observed, implying that both IRES-dependent and scanning-controlled initiation were equally inhibited (Figure 4C,D). This cannot be attributed to the decrease of mRNA levels, since the mRNA expression levels of the bicistronic construct remained similar upon stress exposure (Figure S6). Variations in the transfection efficiency are not likely; transfection efficiency was equal in all experiments as assessed with fluorescent reporter. This suggests that under acute oxidative stress translation of all mRNAs is globally repressed, while moderate oxidative stress affects more strongly the cap-dependent than the IRES-controlled initiation due to the combined effect on eIF2α phosphorylation and the tRNAs deactivation.

## Discussion

Here, we analyze the effect of oxidative stress on the structural integrity of the cellular tRNAs and define the mechanisms of oxidative stress-induced global repression of translation at the level of elongation. Our observations clearly suggest a sequential order of tRNA deactivation upon exposure to oxidative stress: the 3′-terminal CCA sequence is first targeted, while the deactivation into tRNA halves occurs much later. The first event, the CCA cleavage, is not restricted to specific tRNAs; the CCA ends of all tRNAs can be targeted by angiogenin. At severe oxidative stress (500 µM) all tRNAs are rapidly deactivated which leads to a global repression of translational elongation of both mRNAs with scanning and non-scanning (IRES) controlled initiation. The deactivation of the 3′-CCA ends is a mechanism to reversibly repress translation at very low metabolic costs; the 3′-CCA tRNA ends are quickly repaired by the CCA-adding enzyme [8], [10], [30] and translation is reset. In contrast, cleavage in the anticodon loop proceeds on a much slower timescale and is specific for only a subset of tRNAs, so that tiRNAs with specific primary sequences can be generated. This mirrors the reported specificity of the tiRNAs to either selectively target translation of a defined fraction of mRNAs [14] or trigger formation of stress granules [31]. The role of some tiRNAs to silence specific functions [14] indirectly suggests a separation of the tRNA halves upon a cleavage in the anticodon loop. Regeneration of such tRNAs is more metabolically demanding for the cell, as the cleaved tRNAs can be replaced only through a new transcription cycle. A sizeable fraction of tRNAs with cleaved anticodons may not dissociate into tiRNA halves and undergo a repair by tRNA ligases [32]. In vertebrates, the tRNA-ligase activity is coupled to tRNA splicing and is mainly localized in the nucleus [33], [34]; a cytoplasmic localization, although conceivable given the observation for cytoplasmic mitochondrial surface in yeast and plants [35], [36], has not yet been described. This, in turn, would require a translocation of the cleaved tRNAs into the nucleus. Stress-induced retrograde translocation to the nucleus has been shown for mature tRNAs [19]. If upon cleavage within the anticodon the tRNA structure is maintained nearly to the native one, the retrograde transport into the nucleus would be possible and tRNAs can be repaired by the tRNA ligases.

Arsenite derived oxidative stress has also been shown to induce elevated tRNA misacylation with methionine [37]. However, Met-misacylation was obtained upon 1 µM arsenite which is much lower than the concentrations in the experiments presented here (100 or 500 µM). Furthermore, the duration of the treatment is much longer (4 hours) [37], which exceeds the time of the first response towards oxidative stress – the 5′-CCA-ends cleavage. Met-misacylation has been proposed to potentially serve as a protective mechanism for cell's own proteins against oxidative inactivation [37]. This mechanism is distinct from the 3′-CCA cleavage which is useful to regulate global translation activity.

Our observation for selective translation of transcripts with scanning-independent initiation under moderate oxidative stress (100 µM arsenite) adds another layer to selectively reprogram protein translation under stress at the level of elongation. The inhibition of translation initiation through eIF2α phosphorylation upon oxidative stress is a potent mechanism to repress translation of mRNAs with scanning-controlled initiation [3], [16]. At low doses of arsenite (100 µM arsenite) translation of mRNAs with cap-dependent translation initiation is compromised, while the non-scanning, IRES-dependent translation continues to function to a certain level (Figure 4) as in the cell only a small fraction of the total tRNA pool is with deactivated 3′-CCA ends (Figure 1B). Finally, as many transcripts involved in proliferation, differentiation and apoptosis [16] are initiated in a cap-independent manner, the observed differential inactivation of protein synthesis which allows these mRNAs to bypass the global translational repression and activate the selective stress response [3], [16], [38].

## Materials and Methods

### *In vivo* experiments and total tRNA isolation

HeLa cells were usually cultured in DMEM with 10% fetal bovine serum and L-Glu (2 mM) to 80–90% confluency. Oxidative stress was exerted by adding 100 or 500 µM sodium arsenite (Fluka) for indicated times. Human angiogenin was cloned in pCDNA3 plasmid (Invitrogen) and transfected in sub-confluent HeLa cells using polyethylenimine (PEI, Polysciences Europe GmbH). After 8 h angiogenin expression was detected with polyclonal antibodies (1∶1000, Santa Cruz Biotechnology). To decrease the expression of CCA-adding enzyme, pSuper plasmid (Oligoengine) bearing shRNA (5′-CCGGCGCAGAGATCTCACTATAAATCTCGAGATTTATAGTGAGATCTCTGCGTTTTTG-3′) that targeted the CCA-adding enzyme mRNA was transfected using polyethylenimine and expressed for 12 h. shRNA with the same, but randomly scrambled sequence was used as a control. Prior to harvesting, an aliquot of cells was additionally exposed to 500 µM sodium arsenite for 1 h. mRNA was quantified by real-time qRT-PCR and the protein level with polyclonal antibodies (1∶200, Santa Cruz Biotechnology) against human CCA-adding enzyme.

Statistical analyses were performed with Fisher's exact test. Differences were considered statistically significant when *p*<0.05.

A bicistronic construct was created by cloning renilla luciferase (Rluc) gene under the CMV promoter and downstream of it a firefly luciferase (Fluc) gene under the cricket paralysis virus IRES (CrPV-IRES) into pECFP-C1 (Clontech); note CFP was deleted from pECFP-C1 prior to cloning. HeLa cells were transfected with this bicistronic reporter construct using polyethylenimine (PEI, Polysciences Europe GmbH) and expressed for 8 h in total. Prior to harvesting cells were exposed to 100 and 500 µM sodium arsenite for various times and harvested. Luciferase activities were measured using *Dual-Luciferase*® Reporter Assay System (Promega).

For isolation of non-charged tRNAs, HeLa cells were harvested by mechanical scrapping and total RNA was isolated with TriReagent (Sigma-Aldrich) according to the manufacturer's protocol. For isolation of charged tRNAs, total RNA was isolated under acidic conditions. Briefly, HeLa cells were re-suspended in 0.3 M NaOAc 10 mM EDTA pH 4.5 and extracted two times with acidic phenol. The aqueous phase, containing RNA, was precipitated with one volume isopropanol and washed with 80% ethanol. The total uncharged or charged tRNAs were separated on 10% PAGE gels at 4°C. Bands corresponding to the tRNAs were visualized by UV-shadowing, cut and eluted from the gel overnight at 4°C, for uncharged tRNAs in elution buffer (50 mM potassium acetate, 200 mM potassium chloride, pH 7.0) or for charged tRNAs in acidic elution buffer (0.3 M NaOAc, 10 mM EDTA pH 4.5).

### Polysome profiling

1.5 Mio. HeLa cells were treated for 30 min with 100 or 500 µM arsenite. Ten minutes prior to harvesting, cycloheximide (CHX) to a final concentration of 100 µg/ml was added to the medium. Cells were trypsinized (trypsin solution was also supplemented with CHX) and collected by centrifugation at 232×g for 5 min. The cell pellet was resuspended in 320 µl of ice-cold lysis buffer (10 mM Tris-HCl pH 7.4, 5 mM MgCl_2_, 100 mM KCl, 1% Triton-X, 100 µg/ml, 2 mM DTT) and cells were sheared with a 26-gauge syringe. After pelleting of the debris at 5000×g for 8 min at 4°C, the supernatant was layered onto 15 to 50% (w/v) sucrose gradient (20 mM HEPES-KOH pH 7.4, 5 mM MgCl_2_, 100 mM KCl, 100 µg/ml CHX, 2 mM DTT) and centrifuged for 1.5 h at 35,000 rpm in SW 55Ti rotor (Beckman) at 4°C. The gradient was slowly pumped out from the bottom of the tubes and A_254 nm_ was recorded via a flow-through UV spectrophotometer cell (Pharmacia LKB-UV-M II).

### Total mRNA extraction and quantitative RT-PCR

One µg of the total mRNA from HeLa cells was treated with DNase I (Fermentas), the cDNA was synthesized with reverse transcriptase using oligo-dT primer (both Fermentas) and quantified using the 2× Fast SYBR® Green Master Mix (Applied Biosystems) and the 7500 Fast Real-Time PCR system (Applied Biosystems). The following primers were used for amplification of the bicistronic Fluc-Rluc construct: forward (5′-GCTGTTTCTGAGGAGCCTTC-3′) and reverse (5′-GCACTCTGATTGACAAATACGATT-3′), and for CCA-adding enzyme: forward (5′-GATCGCAAAAGAGGAGAAAAAC-3′) and reverse (5′-GCATCAGGTTCCCTAGAATC-3′). mRNA expression was normalized to β-actin.

### Charging assay

The degree of aminoacylation of isolated HeLa tRNAs was tested using the periodate protection assay described previously [39]. Total tRNA sample was treated with 50 mM sodium periodate, which oxidizes the 3′-ends of uncharged tRNAs, which prevents the ligation of the fluorescent stem-loop DNA/RNA oligonucleotide. tRNAs are resolved on denaturing 10% PAGE and the ligation efficiency serves as a measure for the levels of charged tRNAs.

### tRNA microarrays

tRNA probes covering the full-length sequence of 42 cytosplasmic tRNA species with sequences described previously [22] were spotted onto amino-coated slides. The probes for each tRNA are arranged in clusters of six replicates. Radioactively labeled tRNA samples were mixed with 0.17 mg/ml salmon sperm DNA (Invitrogen), 0.17 mg/ml polyA (Sigma-Aldrich) in hybridization buffer (Sigma-Aldrich) and hybridized on the microarrays for 16 h at 60°C. Subsequently, the microarrays were washed three times in 6×SSC at 35°C and once in 2×SSC and 0.2×SSC at 30°C. The composition of the 20×SSC buffer was as follow: 3 M sodium chloride, 300 mM sodium citrate, 0.1% SDS. Radioactivity was detected on a FUJI BAS scanner.

### *In vitro* tRNA transcription

Yeast tRNA^Phe^ with intact CCA ends or tRNA^Phe^CC were generated according to the procedure described in [40]. Radioactive, internally labeled transcripts were synthesized in the presence of 3 µCi ^32^P-α-ATP.

### Radioactive 5′-tRNA labeling

Total HeLa tRNA was dephosphorylated with calf intestine alkaline phosphatase (Roche) for 30 min at 37°C. The enzyme was removed by phenol/chloroform extraction and the tRNA was precipitated. Radioactive phosphate was incorporated by T4 polynukleotide kinase (USB) and ^32^P-γ-ATP for 30 min at 37°C. Radioactively labeled RNA was separated on a denaturing 10% PAGE gel, tRNA bands were cut and eluted in the elution buffer (4 h, 25°C).

### Radioactive 3′-tRNA labeling

Total HeLa tRNAs were deacylated for 45 min at 37°C in 0.1 M TrisHCl, pH 9.0 and dephosphorylated with T4 polynucleotide kinase (USB) in the absence of ATP. 3′-CMP was phosphorylated with ^32^P-γ-ATP (30 min, 37°C) using PNK (Fermentas) and ligated to the RNA with T4 RNA ligase (NEB) by incubation over night at 16°C. Radioactively labeled RNAs were separated on a denaturing 10% PAGE gel, tRNA bands were cut and eluted in the elution buffer (4 h, 25°C).

### *In vitro* angiogenin digestion

To prepare the tRNA for subsequent digestions, isolated tRNA was heated at 90°C for 2 min and cooled down at room temperature in 30 mM HEPES 30 mM sodium chloride, pH 7.0 for 3 min. MgCl_2_ and BSA were added to final concentrations of 2 mM and 0.01%, respectively, and further incubated for 5 min at 37°C. Recombinant human angiogenin (R&D systems) was added to a final concentration of 0.2 or 1 µM to the total HeLa or yeast tRNA^Phe^ and incubated at 37°C for the indicated times. In the radioactive experiments, total non-labeled HeLa tRNA was spiked with radioactive 5′- or 3′-labeled tRNA. The reactions were stopped by extraction with phenol/chloroform or adding gel loading buffer (95% formamide, 0.025% SDS, 0.5 mM EDTA, 0.25% (w/v) bromophenolblue, 0.25% (w/v) xylene cyanol) and shock freezing in liquid nitrogen. To test the integrity of the 3′-CCA ends, a fluorescent stem-loop RNA/DNA oligonucleotide, with a sequence described previously [22], was ligated over night at 16°C with T4 DNA ligase (NEB). Full-length tRNA and tiRNAs were separated on a denaturing 10% PAGE gel.

### *In vitro* repair of the CCA termini with CCA-adding enzyme

Human CCA-adding enzyme was purified as described [41]. Total HeLa tRNA and 3′-radioactive labeled yeast tRNA^Phe^ (0.5 µM) were treated with 0.2 µM angiogenin at 37°C for 4 h, dephosphorylated with PNK to convert the 2′,3′-cyclophosphate generated by angiogenin to 3′-OH [40] and subsequently treated with 50 nM human CCA-adding enzyme at 30°C for 30 min in 20 mM HEPES pH 7.6, containing 20 mM KCl, 6 mM MgCl_2_, 2 mM DTT and 1 mM NTPs.

### Determination of the integrity of the CCA termini of tRNAs

Oxidative stress was exerted by adding 500 µM sodium arsenite (Fluka) to confluent HeLa cells for indicated times. RNA was isolated using mirVana miRNA Isolation kit (Ambion) and subsequently deacylated in 0.1 M Tris.HCl buffer, pH 9.0 at 37°C for 30 min. Fluorescent stem-loop RNA/DNA oligonucleotide [22] (Figure 1B, schematic inset) was ligated over night at 16°C with T4 DNA ligase (NEB). Ligation efficiency was analyzed by resolving the samples on denaturing 10% PAGE and detected by fluorescence (Fujifilm LAS-4000) or SYBR Green (Invitrogen) staining.

## Supporting Information

Figure S1 tRNAs bearing CA-motif in the anticodon are specifically cleaved by angiogenin to tiRNAs. (A) Microarray of tiRNAs derived from 5′ radioactively labeled HeLa tRNAs treated with angiogenin *in vitro*. Full sequences of the probes spotted on the microarray are listed in [22]; the probes for each tRNA are arranged in clusters of six replicates. Spotting scheme of the tRNA probes (bottom panel). tRNAs with a CA-motif in the anticodon loop cleaved by angiogenin into tiRNAs are highlighted in dark green. Not all CA-motif-bearing tRNAs served as angiogenin substrates (light green) most likely due to secondary modifications or low concentrations below the detection limit. tRNAs with CG- and UA-motifs in the anticodon loop which are cleaved by angiogenin are marked in orange; the intact tRNAs in red. (B) Schematic distribution of the CA-motifs in the single-stranded loops of the tRNAs. Single-stranded regions (highlighted in gray) were aligned and the percentage of the tRNAs with CA motifs within the whole tRNA set for one amino acid is represented on the schematic. tRNA sequences are extracted from http://gtrnadb.ucsc.edu/. It should be noted that in the anticodon loop the CA-sequence can be at any position not only within the anticodon.
(TIF)

Figure S2 Alterations in the cellular levels of angiogenin and CCA-adding enzyme. (A) Overexpression of angiogenin at different times. Note that longer expression significantly changed the morphology of the cells. Equal amounts of cells were loaded on the gel as evidenced by the equal intensity of the β-actin (ACTB) band. The numbers on the left denote the molecular mass marker in kDa. Down-regulation of CCA-adding enzyme with siRNA decreases significantly the mRNA (B) but has no impact on the protein level (C). (B) mRNA was quantified by real-time qRT-PCR 12 h after downregulating the CCA-adding enzyme with a specific siRNA probe (siRNA-CCA). Values were normalized to β-actin mRNA for each experiment and expressed as a fold change (log_2_; mean ± SD of three independent experiments) compared to control cells transfected with shRNA with the same, but randomly scrambled sequence. (C) The amount of CCA-adding enzyme was quantified by western blot 12 h after the siRNA-induced downregulation of the expression of CCA-adding enzyme (siRNA-CCA) and subsequent exposure to 500 µM arsenite for 60 min (siRNA-CCA+arsenite). Cells expressing the negative control, siRNA with the randomly scrambled sequence (siRNA-control), were treated the same way. Equal amounts of cells were loaded on the gel as evidenced by the comparable intensity of the β-actin (ACTB) band. The numbers on the left denote the molecular mass marker in kDa.
(TIF)

Figure S3 3′-CCA sequence is removed with a similar kinetics for all tRNAs. Quantification of the microarray analysis in Figure 2C. The intensities of the full-length 3′-labeled tRNAs are represented as a mean intensity ± SD for the 6 replicates of each tRNA.
(TIF)

Figure S4 Angiogenin cleaves only the 3′-terminal adenine residue from the 3′-CCA sequence. Five pmol *in vitro* transcribed yeast tRNA^Phe^ with intact 3′-CCA terminus (A) or truncated 3′-CC end, tRNA^Phe^CC, (B) were incubated with 0.2 µM angiogenin at 37°C. Angiogenin removes selectively the 3′-adenine from intact tRNA^Phe^ (A) while tRNA^Phe^CC remained unchanged (B). tRNA^Phe^CC lacking the terminal 3′-adenosine at the 3′-CCA end served as a standard. Note that tRNA^Phe^ has no CA-motif in the anticodon loop and is not cleaved by angiogenin into tiRNA (Figure S1). tRNA^Phe^ has a very similar 3D-structure to the *in vivo* transcribed tRNA [10], [11] and represents a standard substrate for *in vitro* processing and aminoacylation reactions [12], [13]. (C) The activity of CCA-adding enzyme is insensitive to arsenite. Purified human CCA-adding enzyme (+CCA) was incubated in the presence of different concentrations of arsenite and its activity was tested by repairing the 3′-end of 5 pmol tRNA^Phe^CC lacking the 3′-terminal adenosine. At all arsenite concentrations CCA-adding enzyme added an adenosine to tRNA^Phe^CC completing it to full-length tRNA^Phe^. Arsenite did not alter the structure of the tRNA^Phe^CC itself (see samples with no CCA).
(TIF)

Figure S5 Susceptibility of 3′-CCA ends is independent of the charging status of tRNAs. Five pmol of acylated (A) or deacylated (B) HeLa tRNAs were incubated with 0.2 µM angiogenin for various times. Intact tRNAs that ligate the fluorescent stem-loop oligonucleotide decreased similarly for the acylated (upper gel) and deacylated (lower gel) tRNAs. No ANG, sample was incubated in the reaction buffer without angiogenin and served as a control to monitor unspecific cleavage or changes during incubation. The numbers on the left denote the DNA ladder in nt. More than 90% of the tRNAs isolated from the cell under acidic conditions (A) were charged (compare the samples after periodate treatment from (A) and (B), +IO_4_). Only uncharged tRNAs are oxidized by periodate and are thereafter unable to ligate to the fluorescent stem-loop oligonucleotide. +NaCl, denotes control samples incubated in the reaction buffer with NaCl instead of periodate.
(TIF)

Figure S6 Exposure to arsenite has no effect on mRNA level. Quantification by real-time qRT-PCR of mRNA levels of the ectopically expressed Rluc-Fluc construct in HeLa cells exposed to 100 µM and 500 µM arsenite for different times. Values were normalized to β-actin mRNA for each cell and expressed as a fold change (log_2_; mean ± SD of three independent experiments) compared to the untreated (+DMSO) cells. Differences in the expression level are insignificant (*p*>0.05).
(TIF)
