# Supplementary figures and images for: Reversible and Rapid Transfer-RNA Deactivation as a Mechanism of Translational Repression in Stress
Source: PLoS Genet. 2013 Aug 29;9(8):e1003767. doi: 10.1371/journal.pgen.1003767 (PMC3757041; doi:10.1371/journal.pgen.1003767)

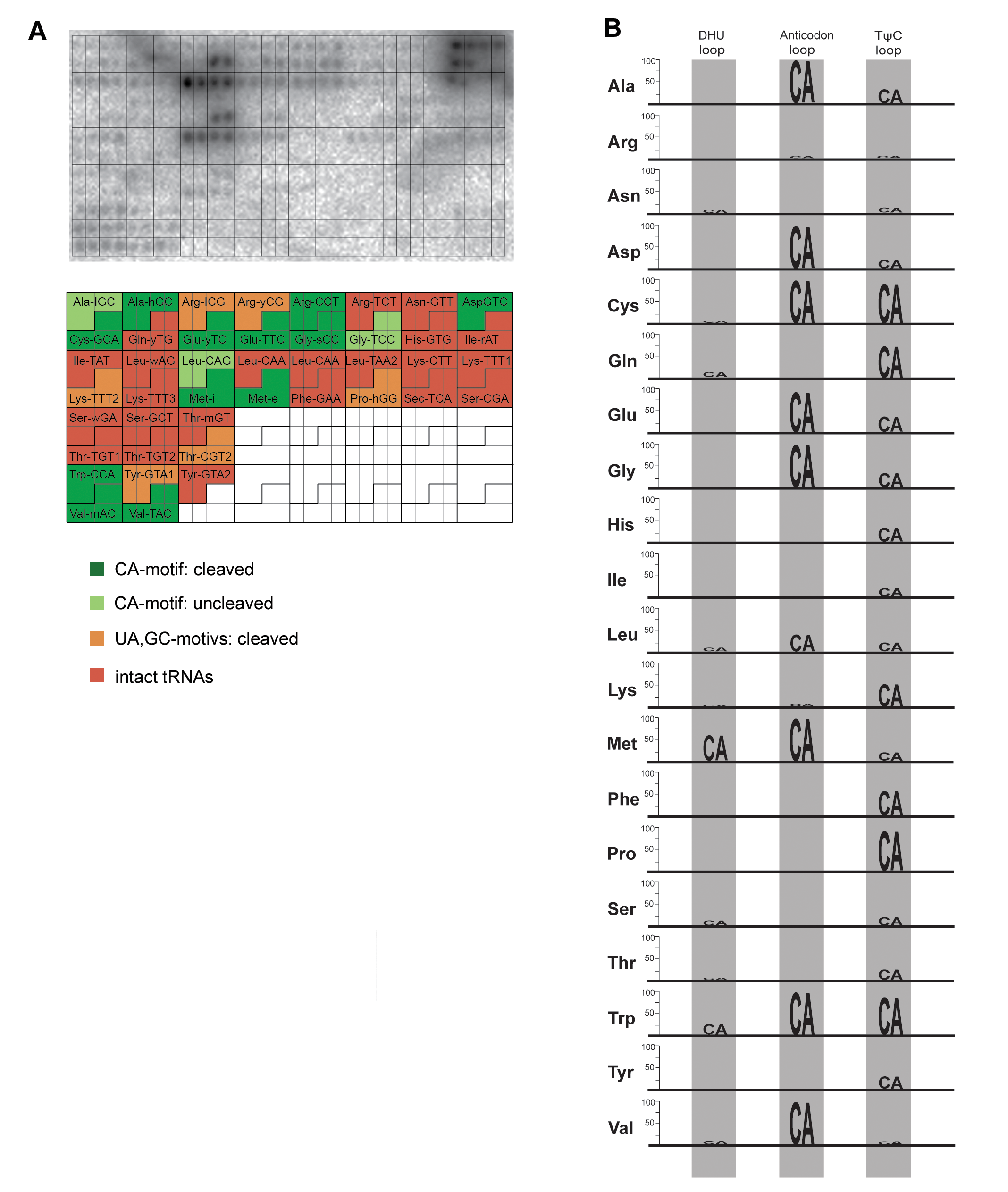

Supplement: Figure S1 — tRNAs bearing CA-motif in the anticodon are specifically cleaved by angiogenin to tiRNAs. (A) Microarray of tiRNAs derived from 5′ radioactively labeled HeLa tRNAs treated with angiogenin in vitro. Full sequences of the probes spotted on the microarray are listed in [22]; the probes for each tRNA are arranged in clusters of six replicates. Spotting scheme of the tRNA probes (bottom panel). tRNAs with a CA-motif in the anticodon loop cleaved by angiogenin into tiRNAs are highlighted in dark green. Not all CA-motif-bearing tRNAs served as angiogenin substrates (light green) most likely due to secondary modifications or low concentrations below the detection limit. tRNAs with CG- and UA-motifs in the anticodon loop which are cleaved by angiogenin are marked in orange; the intact tRNAs in red. (B) Schematic distribution of the CA-motifs in the single-stranded loops of the tRNAs. Single-stranded regions (highlighted in gray) were aligned and the percentage of the tRNAs with CA motifs within the whole tRNA set for one amino acid is represented on the schematic. tRNA sequences are extracted from http://gtrnadb.ucsc.edu/. It should be noted that in the anticodon loop the CA-sequence can be at any position not only within the anticodon. (TIF) [file pgen.1003767.s001.tif]

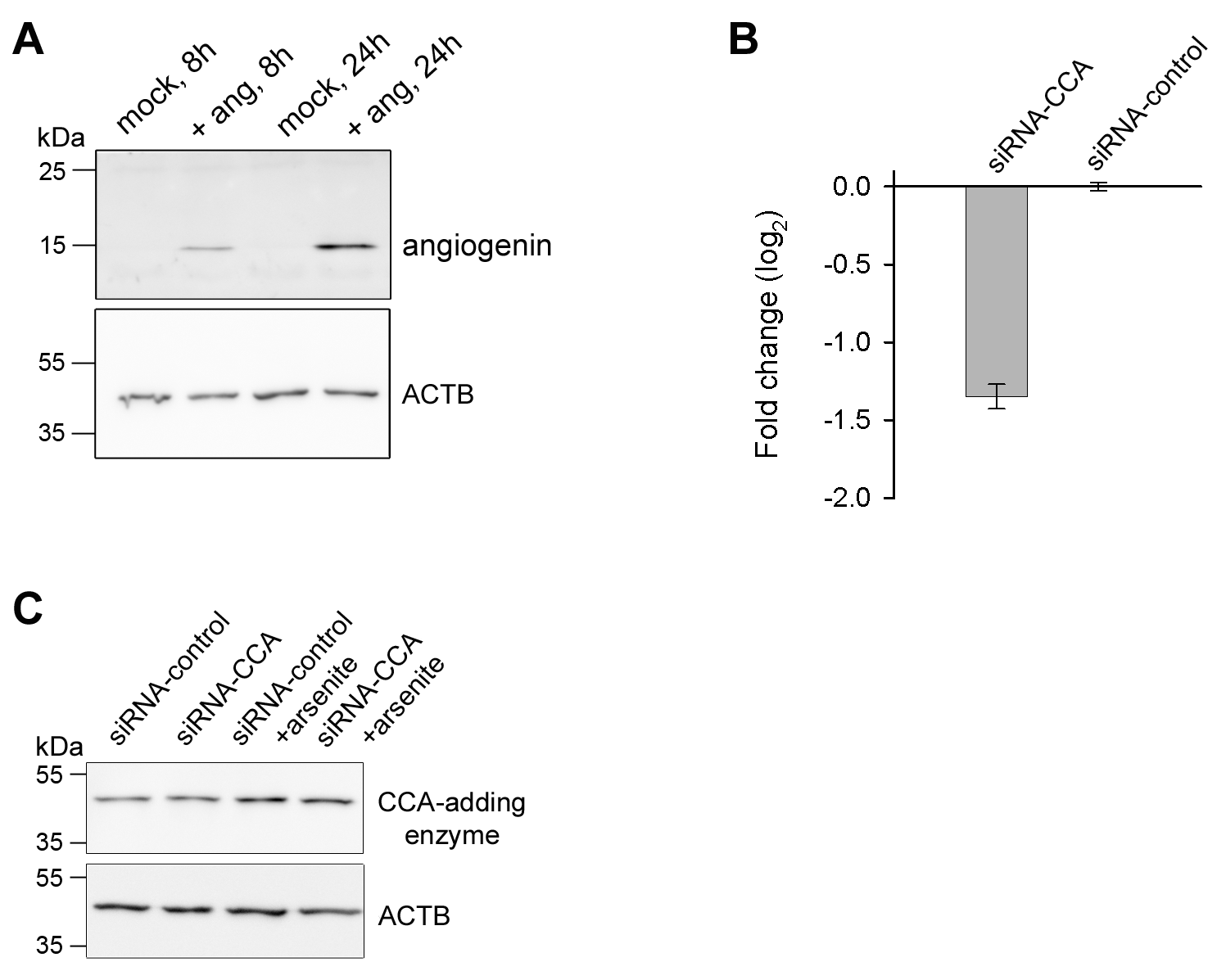

Supplement: Figure S2 — Alterations in the cellular levels of angiogenin and CCA-adding enzyme. (A) Overexpression of angiogenin at different times. Note that longer expression significantly changed the morphology of the cells. Equal amounts of cells were loaded on the gel as evidenced by the equal intensity of the β-actin (ACTB) band. The numbers on the left denote the molecular mass marker in kDa. Down-regulation of CCA-adding enzyme with siRNA decreases significantly the mRNA (B) but has no impact on the protein level (C). (B) mRNA was quantified by real-time qRT-PCR 12 h after downregulating the CCA-adding enzyme with a specific siRNA probe (siRNA-CCA). Values were normalized to β-actin mRNA for each experiment and expressed as a fold change (log2; mean ± SD of three independent experiments) compared to control cells transfected with shRNA with the same, but randomly scrambled sequence. (C) The amount of CCA-adding enzyme was quantified by western blot 12 h after the siRNA-induced downregulation of the expression of CCA-adding enzyme (siRNA-CCA) and subsequent exposure to 500 µM arsenite for 60 min (siRNA-CCA+arsenite). Cells expressing the negative control, siRNA with the randomly scrambled sequence (siRNA-control), were treated the same way. Equal amounts of cells were loaded on the gel as evidenced by the comparable intensity of the β-actin (ACTB) band. The numbers on the left denote the molecular mass marker in kDa. (TIF) [file pgen.1003767.s002.tif]

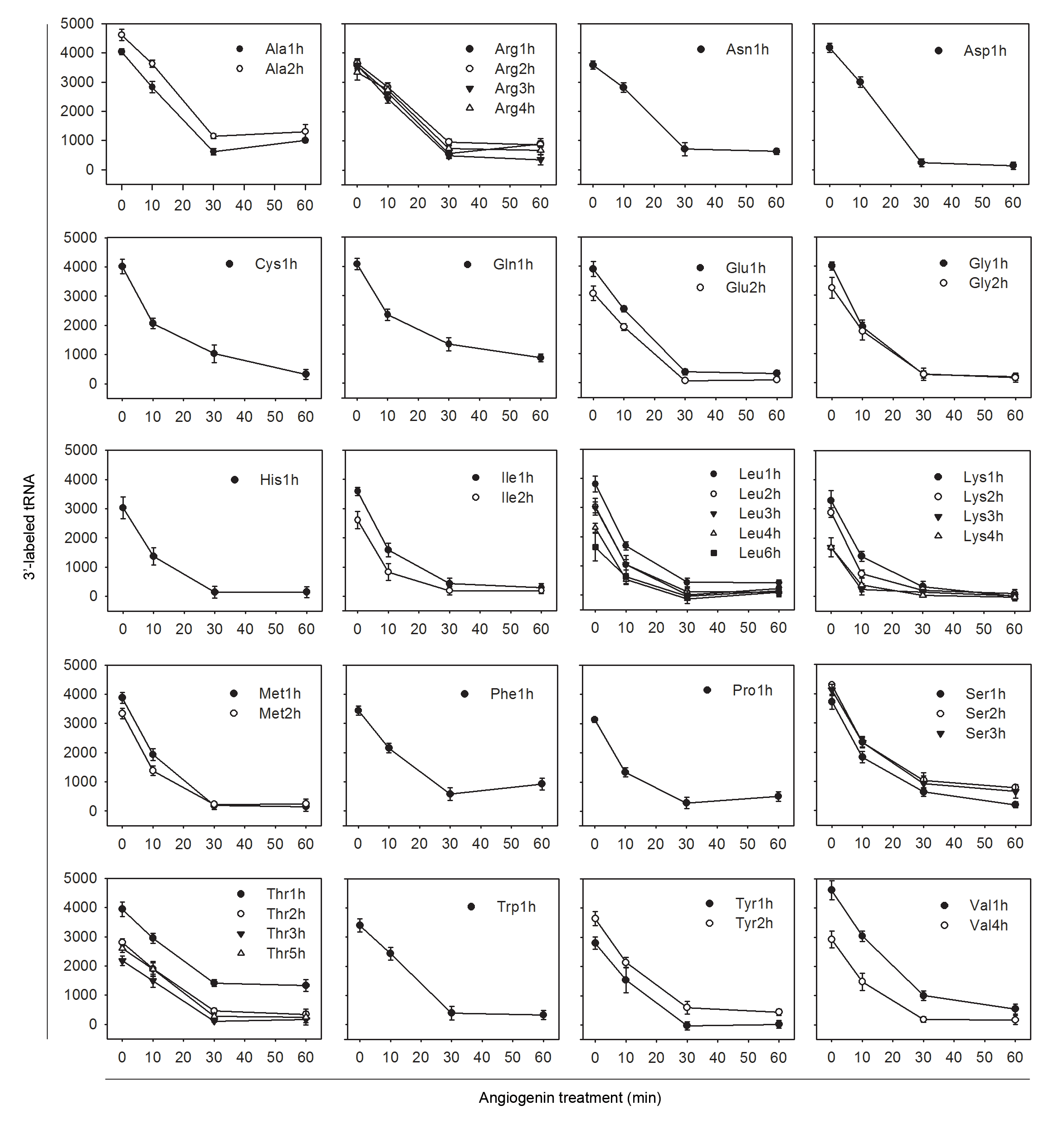

Supplement: Figure S3 — 3′-CCA sequence is removed with a similar kinetics for all tRNAs. Quantification of the microarray analysis in Figure 2C. The intensities of the full-length 3′-labeled tRNAs are represented as a mean intensity ± SD for the 6 replicates of each tRNA. (TIF) [file pgen.1003767.s003.tif]

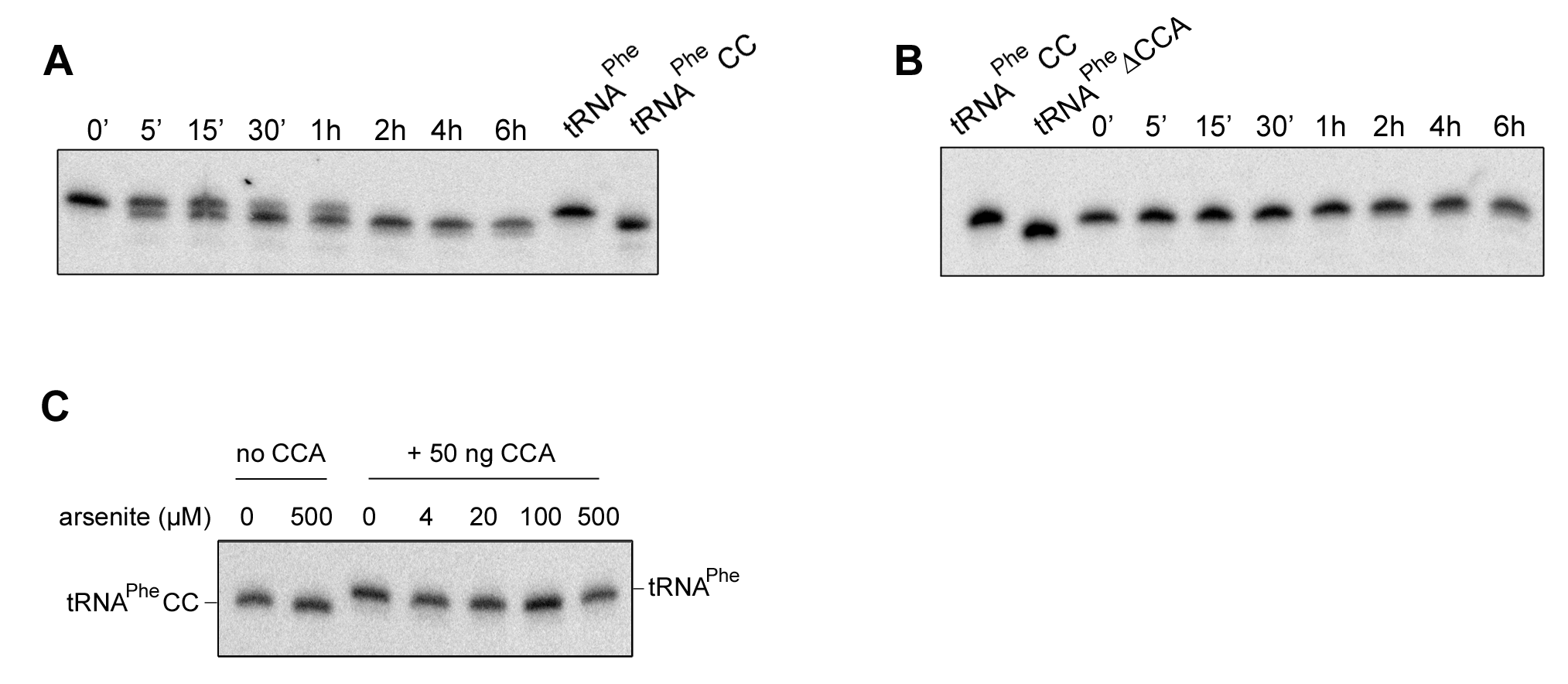

Supplement: Figure S4 — Angiogenin cleaves only the 3′-terminal adenine residue from the 3′-CCA sequence. Five pmol in vitro transcribed yeast tRNAPhe with intact 3′-CCA terminus (A) or truncated 3′-CC end, tRNAPheCC, (B) were incubated with 0.2 µM angiogenin at 37°C. Angiogenin removes selectively the 3′-adenine from intact tRNAPhe (A) while tRNAPheCC remained unchanged (B). tRNAPheCC lacking the terminal 3′-adenosine at the 3′-CCA end served as a standard. Note that tRNAPhe has no CA-motif in the anticodon loop and is not cleaved by angiogenin into tiRNA (Figure S1). tRNAPhe has a very similar 3D-structure to the in vivo transcribed tRNA [10], [11] and represents a standard substrate for in vitro processing and aminoacylation reactions [12], [13]. (C) The activity of CCA-adding enzyme is insensitive to arsenite. Purified human CCA-adding enzyme (+CCA) was incubated in the presence of different concentrations of arsenite and its activity was tested by repairing the 3′-end of 5 pmol tRNAPheCC lacking the 3′-terminal adenosine. At all arsenite concentrations CCA-adding enzyme added an adenosine to tRNAPheCC completing it to full-length tRNAPhe. Arsenite did not alter the structure of the tRNAPheCC itself (see samples with no CCA). (TIF) [file pgen.1003767.s004.tif]

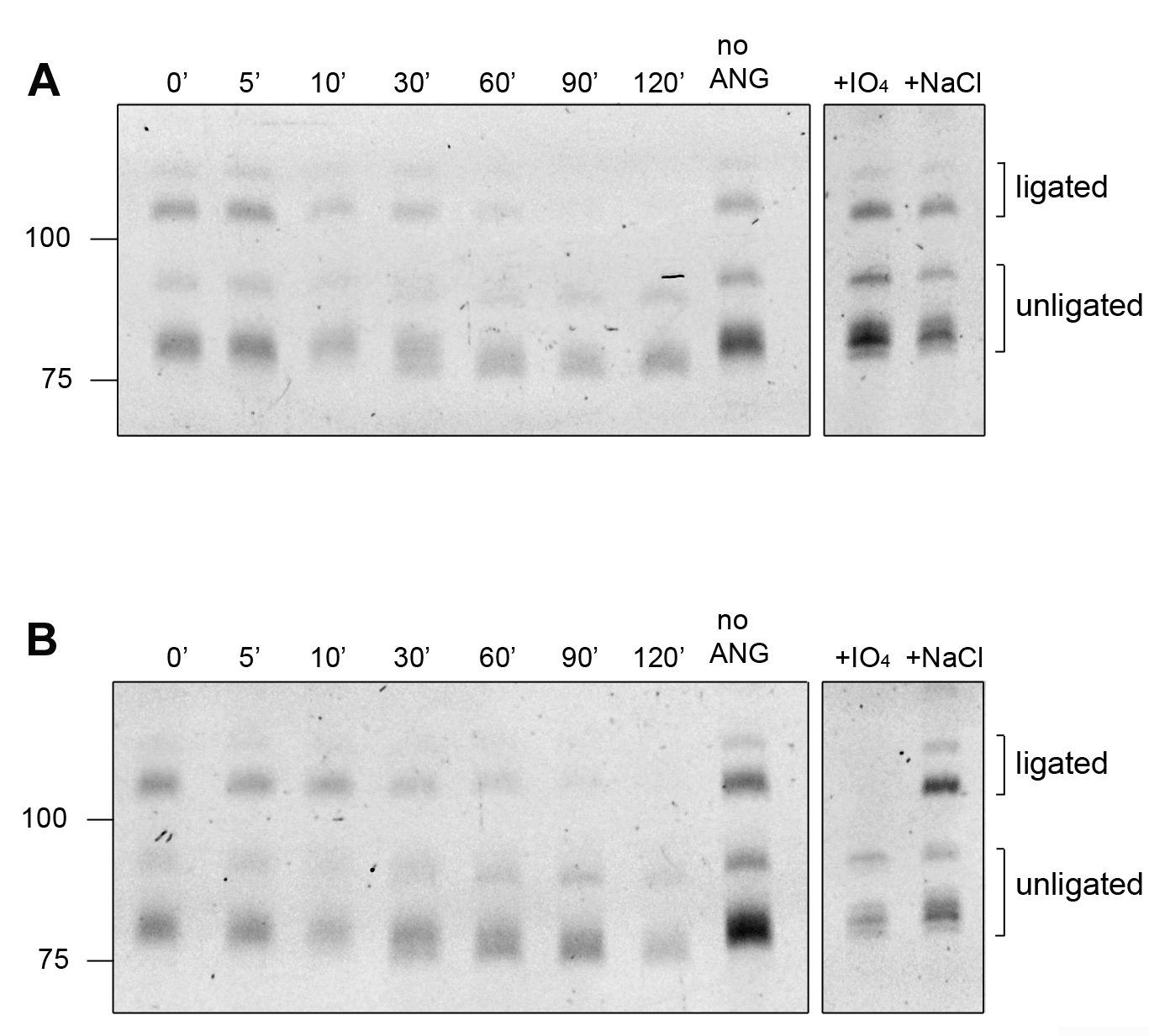

Supplement: Figure S5 — Susceptibility of 3′-CCA ends is independent of the charging status of tRNAs. Five pmol of acylated (A) or deacylated (B) HeLa tRNAs were incubated with 0.2 µM angiogenin for various times. Intact tRNAs that ligate the fluorescent stem-loop oligonucleotide decreased similarly for the acylated (upper gel) and deacylated (lower gel) tRNAs. No ANG, sample was incubated in the reaction buffer without angiogenin and served as a control to monitor unspecific cleavage or changes during incubation. The numbers on the left denote the DNA ladder in nt. More than 90% of the tRNAs isolated from the cell under acidic conditions (A) were charged (compare the samples after periodate treatment from (A) and (B), +IO4). Only uncharged tRNAs are oxidized by periodate and are thereafter unable to ligate to the fluorescent stem-loop oligonucleotide. +NaCl, denotes control samples incubated in the reaction buffer with NaCl instead of periodate. (TIF) [file pgen.1003767.s005.tif]

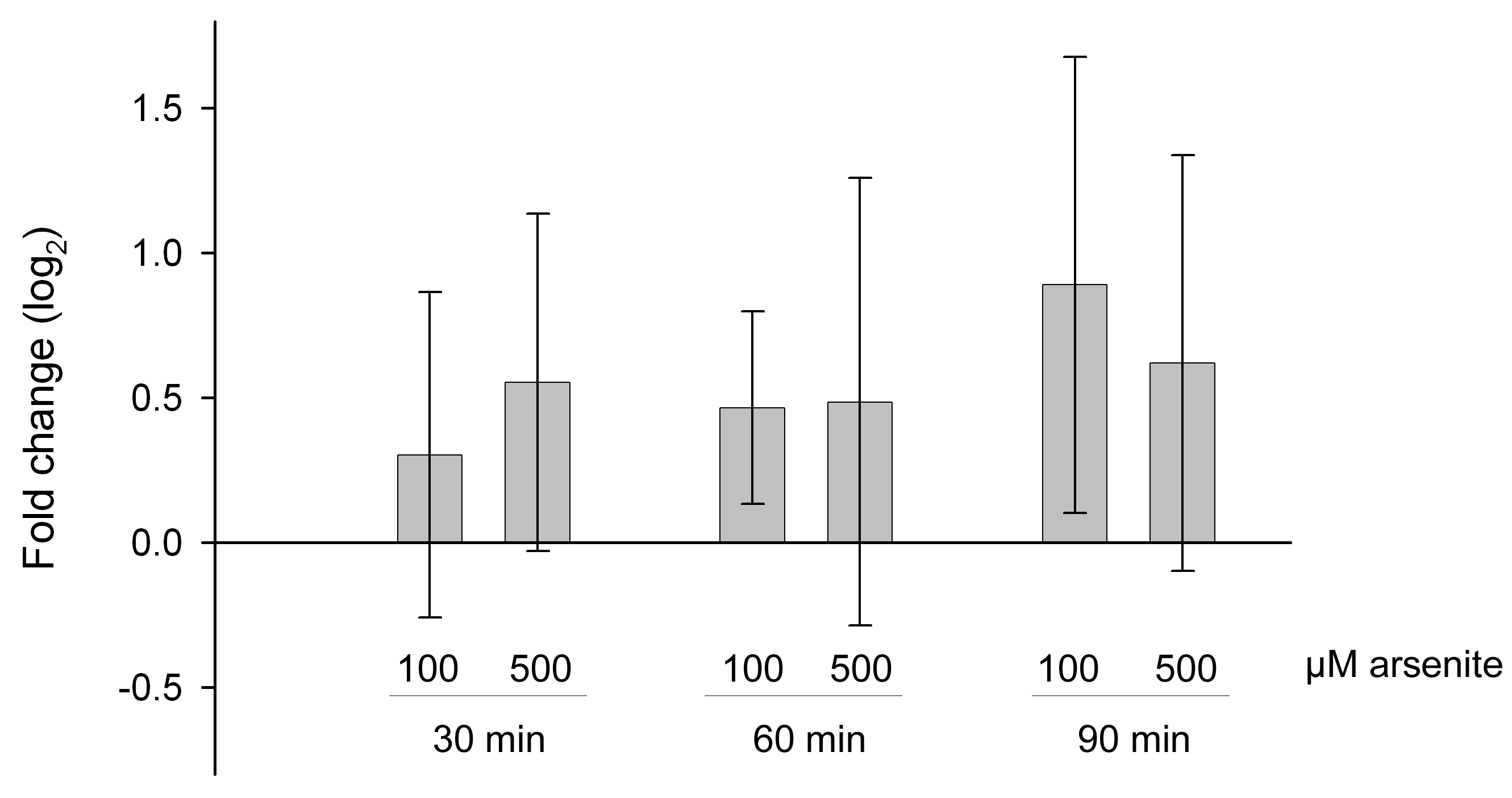

Supplement: Figure S6 — Exposure to arsenite has no effect on mRNA level. Quantification by real-time qRT-PCR of mRNA levels of the ectopically expressed Rluc-Fluc construct in HeLa cells exposed to 100 µM and 500 µM arsenite for different times. Values were normalized to β-actin mRNA for each cell and expressed as a fold change (log2; mean ± SD of three independent experiments) compared to the untreated (+DMSO) cells. Differences in the expression level are insignificant (p>0.05). (TIF) [file pgen.1003767.s006.tif]
